# Editorial: Precision medicine for acute myeloid leukemia

**DOI:** 10.3389/fonc.2023.1233757

**Published:** 2023-07-25

**Authors:** Giovanni Marconi, Fabio Guolo, Jacopo Nanni, Cristina Papayannidis

**Affiliations:** ^1^ IRCCS Istituto Romagnolo per lo Studio dei Tumori (IRST) “Dino Amadori”, Meldola, Italy; ^2^ Chair of Hematology, Department of Internal Medicine (DiMI), University of Genoa, Genova, Italy; ^3^ Clinica Ematologica, Dipartimento di Oncologia e Ematologia (DIPOE), IRCCS Ospedale Policlinico San Martino, Genova, Italy; ^4^ Dipartimento di Scienze Mediche e Chirurgiche, Istituto di Ematologia “Seràgnoli”, Università degli Studi di Bologna, Bologna, Italy; ^5^ IRCCS Azienda Ospedaliero-Universitaria di Bologna, Istituto di Ematologia “Seràgnoli”, Bologna, Italy

**Keywords:** leukemia (acute myeloid), precision medicine, personalized therapy, target agents, new therapeutic agents

## Introduction

Acute myeloid leukemia (AML) has a poor prognosis due to factors like advanced age, high relapse rates, and resistance to standard treatments. Personalized medicine plays a critical role in AML by tailoring treatment approaches to the individual characteristics of each patient, such as their genetic mutations, chromosomal abnormalities, and overall health status, potentially leading to improved treatment outcomes. In this Research Topic, personalized medicine approaches were explored in different scenarios.

## Research of novel biomarkers

Landmark investigations have identified specific genetic mutations and chromosomal abnormalities that play a central role in AML development (1, 2). This knowledge has led to a molecular-based subclassification system, enabling precision medicine in AML treatment.

In this view, Zhang et al. explored a novel putative prognostic marker in children. Transcriptomics analysis was performed on an mRNA dataset from 27 children with non-M3 AML, and genes with the top 5000 median absolute deviation values were selected for further analysis. Two gene modules associated with AML risk groups were identified through enrichment analysis. Survival analysis highlighted FTH1- a gene involved in ferroptosis - as a key gene related to AML prognosis. The study verified the effects of ferroptosis on MOLM-13 and THP-1 cell lines using specific inhibitors, concluding that elevated FTH1 expression promoted cell proliferation and inhibited apoptosis in leukemia cells.

## Optimization of combination chemotherapy approaches

Despite the advancements in the genomic classification of AML, chemotherapy is still the mainstay of AML therapy and is mostly based on combinations of cytarabine and anthracyclines. In this Research Topic, Zhu et al. described the feasibility of a novel therapeutic combination regimen based on multi-modal and multi-agent therapy. This study involved newly diagnosed AML patients who received the decitabine+HAAG (homoharringtonine, aclarubicin, low-dose cytarabine and G-CSF) regimen. The treatment was generally well-tolerated, with manageable toxicities and the vast majority of patients in this study (96%) achieved complete remission after one cycle of induction therapy. Median overall survival was 40 months, and median relapse-free survival was 38 months. Factors such as hematopoietic stem cell transplantation and treatment-related mortality risk were identified as independent prognostic factors for improved outcomes.

## Real life evidence from low-intensity therapies based on hypomethylating agents (HMAs) and venetoclax

Recently, venetoclax has shown promising results in enhancing the effectiveness of HMAs (3–5) and has become standard of care for elderly AML patient ineligible to conventional chemotherapy. However, in this particular population, real life data are very important in order to confirm data from clinical trials, as many of the elderly patients who are treated in the everyday practice may not meet the inclusion criteria for enrollment in clinical trial.

In this Research Topic, Sciumè et al. reported a monocentric real life experience of HMA+Venetoclax. Study population included 60 elderly patients with treatment-naïve (23, 38%) or refractory/relapsed (R/R) AML (37, 62%), who were not eligible for intensive chemotherapy and thus were treated with venetoclax combinations outside of clinical trials. The median age was 70 years, and among the R/R AML patients, 30% had already received an allogeneic stem cell transplantation. The overall response rate was 60%, with 53% achieving complete remission (CR). The CR rate was higher in treatment-naïve AML (78%) compared to R/R AML (49%) (p = 0.017). Median overall survival was 130 days for R/R patients and 269 days for treatment-naïve patients. Measurable residual disease was negative in 26% of evaluable patients in CR/CR with incomplete hematologic recovery after 2 cycles, and in 50% after 4 cycles. In the whole group, 11 patients (18%) underwent allo-HSCT after receiving venetoclax combinations.

Despite being mostly an outpatient treatment, injectable HMAs can be burdensome for patients due to frequent hospital visits. In this view, Delmas et al. reported on the administration preference between different modes of administration (MOA) and the relative importance of treatment-related characteristics in influencing treatment decisions.

Semi-structured interviews were conducted with 21 adult AML patients in Germany, the United Kingdom, and Spain, who were either not eligible for standard chemotherapy, had previous experience with HMAs, or were scheduled for HMA treatment. Patients discussed their experiences with AML and its treatments, and then hypothetical treatment scenarios were presented to assess their preferences. A ranking exercise was also conducted to evaluate the importance of treatment characteristics influencing their treatment decisions for AML. The results showed that the majority of patients (71%) preferred oral administration over parenteral routes due to convenience. Patients who preferred intravenous or subcutaneous routes (24%) cited faster action and onsite monitoring as reasons for their preference. When given a hypothetical choice between two identical AML treatments differing only in MOA, the majority (76%) preferred the oral route. In terms of treatment characteristics influencing decisions, efficacy (86%) and side effects (62%) were most frequently reported as important. Mode of administration, daily life impacts, and treatment location (hospital versus home) were also considered, but to a lesser extent. Efficacy and side effects were rated as the most significant factors in treatment decisions (67% and 19% respectively), while dosing regimen was considered the least important (33%).

## Exploring the role of immunotherapy

Harnessing the immune system against leukemia is a crucial innovation, with agents like anti-CD47 antibody magrolimab, TIM3 inhibitor sabatolimab, and cellular therapies showing potential (6). In this Research Topic, Shahzad et al. reviewed currently available data on the safety and effectiveness of chimeric antigen receptor T-cell (CAR-T) in AML patients. Data were extracted from 13 relevant studies to evaluate the outcomes of CAR-T therapy in relapsed or refractory AML patients. The most commonly targeted antigens in CAR-T therapy for AML were CD33, CD123, NKG2D, and CLL-1. CAR-T therapy had a pooled overall response rate of 65.2% and a complete response rate of 49.5%. The incidence of cytokine release syndrome was 54.4%, while immune-effector cell-associated neurotoxicity syndrome and graft-versus-host disease had lower incidence rates.

## Conclusion

AML treatment is undergoing rapid evolution, employing a range of diverse approaches. Customization of treatment plans, considering clinical factors and the specific biological characteristics of AML, is essential. Personalized approaches have the potential to enhance treatment outcomes and maximize efficacy.

## Author contributions

All the authors equally contributed to the conceptualization, writing and revision of this editorial. All authors contributed to the article and approved the submitted version.

## References

[1] Papaemmanuil E Gerstung M Bullinger L Gaidzik VI Paschka P Roberts ND. Genomic classification and prognosis in acute myeloid leukemia. New Engl J Med (2016) 374:2209–21. doi: 10.1056/NEJMoa1516192 PMC4979995 27276561

[2] Grimwade D Hills RK Moorman AV Walker H Chatters S Goldstone AH. Refinement of cytogenetic classification in acute myeloid leukemia: determination of prognostic significance of rare recurring chromosomal abnormalities among 5876 younger adult patients treated in the United Kingdom Medical Research Council trials. Blood (2010) 116:354–65. doi: 10.1182/blood-2009-11-254441 20385793

[3] DiNardo CD Jonas BA Pullarkat V Thirman MJ Garcia JS Wei AH. Azacitidine and venetoclax in previously untreated acute myeloid leukemia. New Engl J Med (2020) 383:617–29. doi: 10.1056/NEJMOA2012971/SUPPL_FILE/NEJMOA2012971_DATA-SHARING.PDF 32786187

[4] DiNardo CD Lachowiez CA Takahashi K Loghavi S Xiao L Kadia T. Venetoclax combined with FLAG-IDA induction and consolidation in newly diagnosed and relapsed or refractory acute myeloid leukemia. J Clin Oncol (2021) 39(25):2768–78. doi: 10.1200/jco.20.03736 PMC8407653 34043428

[5] Chua CC Roberts AW Reynolds J Fong CY Ting SB Salmon JM. Chemotherapy and venetoclax in elderly acute myeloid leukemia trial (CAVEAT): A phase ib dose-escalation study of venetoclax combined with modified intensive chemotherapy. J Clin Oncol (2020) 38:3506–17. doi: 10.1200/JCO.20.00572 32687450

[6] Ciotti G Marconi G Sperotto A Giannini MB Gottardi M Martinelli G. Biological therapy in elderly patients with acute myeloid leukemia. Expert Opin Biol Ther (2023) 23:175–94. doi: 10.1080/14712598.2023.2174015 36715330

